# Effect of environmental variance-based resilience selection on the gut metabolome of rabbits

**DOI:** 10.1186/s12711-023-00791-5

**Published:** 2023-03-09

**Authors:** Cristina Casto-Rebollo, María José Argente, María Luz García, Agustín Blasco, Noelia Ibáñez-Escriche

**Affiliations:** 1 Institute for Animal Science and Technology, Universitat Politècnica de València, 46022 València, Spain; 2 Centro de Investigación e Innovación Agroalimentaria y Agroambiental (CIAGRO_UMH), Miguel Hernández University, 03312 Orihuela, Spain

## Abstract

**Background:**

Gut metabolites are key actors in host-microbiota crosstalk with effect on health. The study of the gut metabolome is an emerging topic in livestock, which can help understand its effect on key traits such as animal resilience and welfare. Animal resilience has now become a major trait of interest because of the high demand for more sustainable production. Composition of the gut microbiome can reveal mechanisms that underlie animal resilience because of its influence on host immunity. Environmental variance (V_E_), specifically the residual variance, is one measure of resilience. The aim of this study was to identify gut metabolites that underlie differences in the resilience potential of animals originating from a divergent selection for V_E_ of litter size (LS). We performed an untargeted gut metabolome analysis in two divergent rabbit populations for low (n = 13) and high (n = 13) V_E_ of LS. Partial least square-discriminant analysis was undertaken, and Bayesian statistics were computed to determine dissimilarities in the gut metabolites between these two rabbit populations.

**Results:**

We identified 15 metabolites that discriminate rabbits from the divergent populations with a prediction performance of 99.2% and 90.4% for the resilient and non-resilient populations, respectively. These metabolites were suggested to be biomarkers of animal resilience as they were the most reliable. Among these, five that derived from the microbiota metabolism (3-(4-hydroxyphenyl)lactate, 5-aminovalerate, and equol, N6-acetyllysine, and serine), were suggested to be indicators of dissimilarities in the microbiome composition between the rabbit populations. The abundances of acylcarnitines and metabolites derived from the phenylalanine, tyrosine, and tryptophan metabolism were low in the resilient population and these pathways can, therefore impact the inflammatory response and health status of animals.

**Conclusions:**

This is the first study to identify gut metabolites that could act as potential resilience biomarkers. The results support differences in resilience between the two studied rabbit populations that were generated by selection for V_E_ of LS. Furthermore, selection for V_E_ of LS modified the gut metabolome, which could be another factor that modulates animal resilience. Further studies are needed to determine the causal role of these metabolites in health and disease.

**Supplementary Information:**

The online version contains supplementary material available at 10.1186/s12711-023-00791-5.

## Background

Gut metabolites are key actors in host-microbiota crosstalk and can have either beneficial or detrimental effects on the host [1–3]. They can act in the gut or travel through the plasma to reach the host’s tissues, i.e. they can influence the functions of the liver, brain, and immune system [4]. Gut metabolites can be derived from (i) the microbiota, due to the conversion of non-digestible components from the diet or to de novo synthesis, (ii) the host, and (iii) the diet. Metabolites from the host can also be modified by the gut microbiota [5, 6]. In livestock, the study of the gut metabolome is an emerging topic. Recently, differences in the gut metabolome have been found to be associated with traits such as feed efficiency [7, 8] and milk yield protein [9]. Thus, the study of the gut metabolome can help to expand the knowledge on the interactions between the gut and peripheral tissues, and also to understand its effect on key traits such as methane emission and animal resilience.

Animal resilience has become a major trait of interest in livestock because of the high demand for more sustainable livestock systems. Resilience is the ability of animals to maintain or quickly recover their production performance when an environmental perturbation occurs [10]. Since environmental variance (V_E_) is highly correlated with animal resilience, it is an interesting trait for measuring resilience [10–12]. Indeed, animals with a low V_E_ for a given trait seem to cope better with environmental disturbances [11, 12]. Quantitative genetics and genomic studies in different species underline the importance of the immune system in modulating animal resilience [13–16]. The gut metabolome is closely related to the modulation of the immune system [3, 17], thus its study could provide insight into the mechanisms that underlie animal resilience.

A previous metagenomic study found that microbiome composition (genes and species) differs between two rabbit populations that had been selected for high and low V_E_ of litter size (LS) [18]. These two lines showed a notable genetic response to this selection, and also correlated responses in resilience indicators that were measured after a vaccination challenge [12] or immediately following first parity [14]. The present study is an extension of a previous metagenome study [18] and its aim was to identify gut metabolites that are related to the resilience potential of these rabbit populations. The gut microbiome is a complex ecosystem that is strongly influenced by environmental factors [19, 20] and the origin of metabolites [6]. Reducing the impact of confounding factors is necessary to correctly decipher variability in the gut metabolome that underlies complex traits. The rabbit populations used in this study are coetaneous and were selected under the same environmental conditions and diet for 13 generations, and also showed differences in resilience potential [12, 14]. Thus, they constitute an exceptional resource to identify gut metabolites that can act as biomarkers of animal resilience.

## Methods

The rabbits used in this study belonged to the 13th generation of a divergent selection experiment for high and low V_E_ of LS that was carried out at the University Miguel Hernández of Elche (Spain) [21]. Cecum samples were collected from 28 does (14 from each population) that were sacrificed after their first parity by intravenous injection of sodium thiopental at a dose of 50 mg/kg of body weight (Thiobarbital, B. Braun Medical S. A., Barcelona, Spain). The samples were homogenized in 50-mL Falcon tubes and aliquoted in 2-mL cryotubes for immediate snap-freezing in liquid nitrogen and storage at − 80 °C until they were processed.

Untargeted metabolite analysis of the gut content was conducted on the Metabolon Discovery HD4 platform. The samples were prepared by the Hamilton Company's automated MicroLab STAR® system. Prior to extraction, several recovery standards were added for quality control purposes. Proteins in the samples were precipitated with methanol under vigorous shaking for two min, followed by centrifugation to recover chemically diverse metabolites. The resulting extract was divided into five aliquots and the TurboVap® (Zymark) evaporator was used to remove organic solvents.

The metabolites in the gut were profiled by Ultrahigh Performance Liquid Chromatography (UPLC) and Tandem Mass Spectrometry (UPLC-MS/MS) with negative and positive ion mode electrospray ionization (ESI). All methods used a Waters ACQUITY UPLC system (Waters, Milford, MA, USA) and a Q-Exactive high resolution/accurate mass spectrometer (Thermo Fisher Scientific) interfaced with a heated electrospray ionization (HESI-II) source and an Orbitrap mass analyser operated at 35,000 mass resolution. The aliquots were dried and resuspended in solvents that are compatible with the method used and that contain standards at fixed concentrations to ensure injection and chromatographic consistency. Quality control samples were injected throughout the platform run to remove artifacts and background noise and to distinguish biological variability from process/instrument variability.

Among the five aliquots, two were analysed by two separate reverse phase (RP)/UPLC-MS/MS methods with the acidic positive ion mode ESI. One was chromatographically-optimized for more hydrophilic compounds and the other for more hydrophobic compounds. To detect the former, the aliquot was gradient-eluted from a C18 column (Waters UPLC BEH C18-2.1 × 100 mm, 1.7 µm) using a water and methanol solution that contained 0.05% perfluoropentanoic acid (PFPA) and 0.1% formic acid (FA). To detect the latter, the aliquot was gradient-eluted from the same C18 column but using an overall higher organic solution composed of methanol, acetonitrile, water, 0.05% PFPA, and 0.01% FA. The third aliquot was analysed by RP/UPLC-MS/MS with the basic negative ion mode ESI using a separate dedicated C18 column and eluted with methanol, water, and 6.5 mM of ammonium bicarbonate at pH 8. The fourth aliquot was analysed via a negative ion mode ESI with a HILIC column (Waters UPLC BEH Amide 2.1 × 150 mm, 1.7 µm), using a gradient of water and acetonitrile with 10 mM ammonium formate at pH 10.8. The last aliquot was reserved for backup. Raw data files were obtained after tandem mass spectrometry analysis by alternating between MS and data-dependent MS^n^ scans, using dynamic exclusion. The scan range varied slightly between chromatography methods but covered a 70 to 1000 mass to charge ratio (m/z).

Raw data were extracted, peaks were identified, and quality control was processed on the Metabolon hardware and software. In total, 765 metabolites were identified by a library that included three criteria of more than 3300 authenticated standard components: retention time/index (RI), mass to charge ratio (*m/z*), and chromatographic data, including MS/MS spectral data. All three criteria can be used to distinguish and differentiate metabolites. Metabolite quantification was based on the area-under-the-curve of the detected peaks.

All statistical analyses were done in R [22]. A principal component analysis was computed using 480 of the 765 metabolites that had no missing values. Of the 28 animals, 13 animals remained in the datasets from both the low (resilient) and the high (non-resilient) V_E_ of LS populations. Metabolites with more than 20% missing values [23] within each population were considered uninformative and were removed from the dataset. The remaining missing values were replaced by half of the minimum peak intensity identified by the UPLC-MS/MS method to which each metabolite belonged. Due to the compositional nature of metabolomic data [24], the data on 654 metabolites from the 26 samples were transformed using the additive log-ratio (ALR) transformation, as follows:

1 $$ALR\left(\mathrm{j}|ref\right)=\mathrm{log}\left(\frac{{x}_{\mathrm{j}}}{{x}_{ref}}\right)=\mathrm{log}\left({x}_{\mathrm{j}}\right)-\mathrm{log}\left({x}_{ref}\right),$$

where the number of total ALR is $\mathrm{j}$-1, $\mathrm{j}$ being the total number of variables in the dataset and ${x}_{ref}$ is the reference variable (uracil nucleotide) with the lowest coefficient of variation. Procrustes correlation was performed to check for lack of isometry in the transformed dataset [25]. ALR-transformed data were auto-scaled to a mean of 0 and a standard deviation (SD) of 1.

A partial least square-discriminant analysis (PLS-DA) was performed to identify the most relevant metabolites for discriminating rabbits from the resilient and non-resilient populations. The PLS-DA model was computed using the mixOmics package in R [26], using a categorical vector $\mathbf{y}$ that indicates the rabbit population for each sample (high or low V_E_), and a matrix $\mathbf{X}$ with the ALR of each metabolite for each sample. The optimal number of components was that with the lowest balance error rate (BER) for Mahalanobis distance, computed by fourfold cross-validation repeated 100 times. Metabolites that had a contribution to model prediction or variable important prediction (VIP) less than 1 were removed from the dataset, and a new PLS-DA model was computed [27]. PLS-DA model computing and variable selection were performed until the lowest BER was achieved.

The prediction performance of the final model was validated using a confusion matrix and a permuted-confusion matrix. The former was constructed by fourfold cross-validation repeated 10,000 times using the Mahalanobis distance to predict the rabbit populations. The accuracy and precision of the model were calculated considering that the resilient population was the true positive value. We also computed a permuted-confusion matrix by randomizing the categorical $\mathbf{y}$ vector to check whether the prediction performance of the final models was spurious, i.e. whether the percentage of true positives in the permuted-confusion matrix was far from the 50% expected under random assignment of two categories.

Bayesian statistics were used to determine the relevance of the difference in the metabolite abundance between the two rabbit populations using a model that included a single effect for line and flat priors for all unknowns. Marginal posterior distributions of the unknowns were estimated by Monte Carlo Markov chains (Gibbs sampling) using four chains with a length of 50,000 iterations, a lag of 10, and a burn-in of 1000 iterations. The posterior mean of the difference in metabolite abundance was estimated as the mean of the marginal posterior distribution of the difference between the resilient and the non-resilient populations. These differences were quantified in units of SD of each metabolite. The probability of the difference [28] being greater (if the difference is positive) or less (if negative) than 0 (P_0_) was also calculated. To establish a threshold for the identification of relevant metabolites an approximation of the false discover rate (FDR) of Storey [29] was calculated based on the cumulative posterior error probability (PEP), similar to the q-value. The PEP was calculated as (1 − _0_)/0.5. We assumed a cumulative PEP of 0.05, meaning that approximately 5% of the relevant metabolites identified were allowed to be false positives. Then, we performed an analysis for assigning the biological origin of each relevant metabolite using the metOrigin tool [30]. A full record of the method used is in Additional file 1.

## Results

**Fig. 1:**
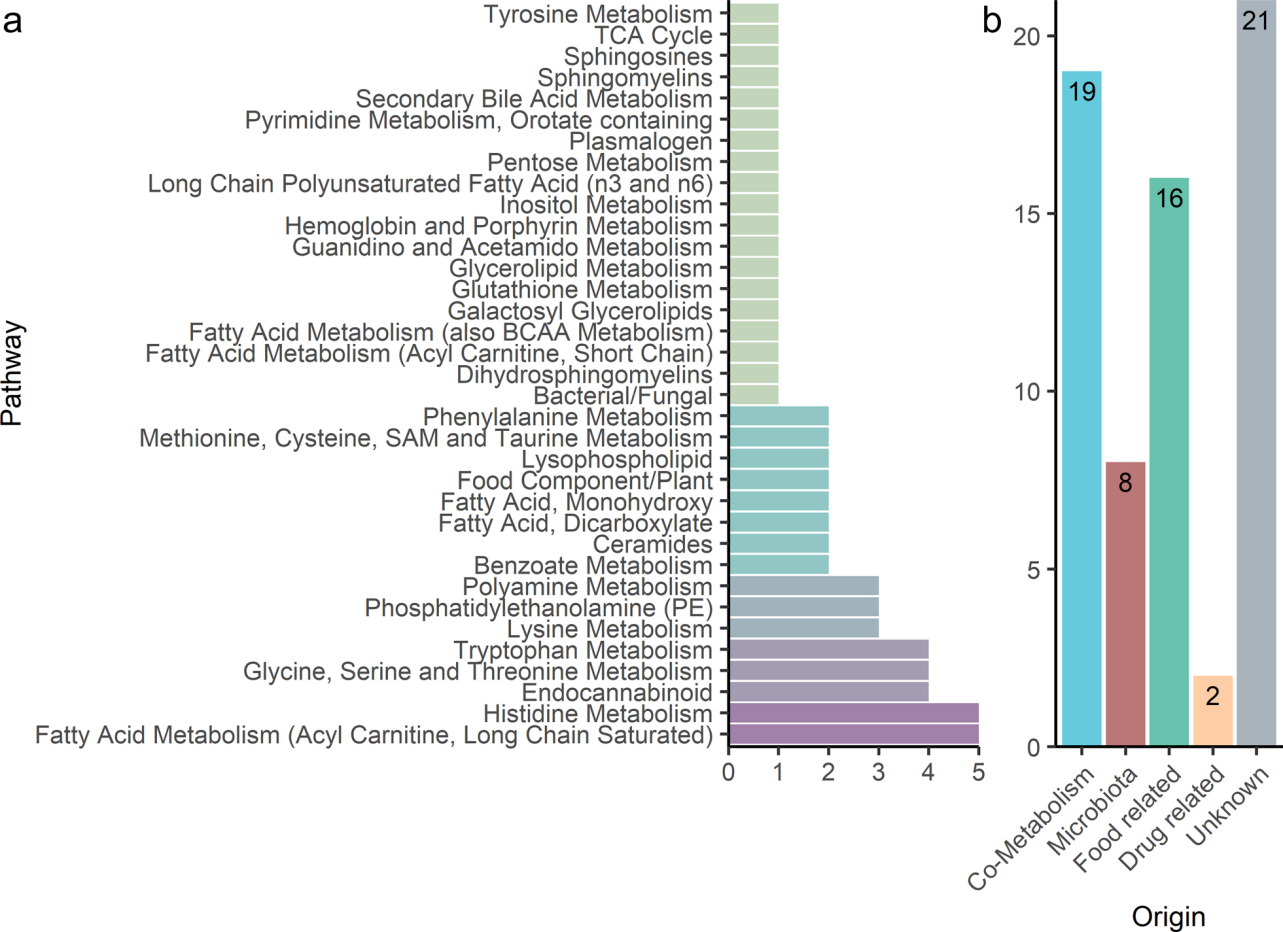
Pathway and biological origin of metabolites with a relevant difference in abundance between the divergent rabbit populations. **a** Pathways of the 66 metabolites identified with a relevant difference in abundance between the resilient and non-resilient rabbit populations. **b** Biological origin of the 66 metabolites with a relevant difference in abundance between the two rabbit populations. “Co-metabolism” refers to metabolites that are shared between the host and the microbiota. “Microbiota” are microbiota-derived metabolites. “Food related” are metabolites obtained from the diet. “Drug” refers to drug-related metabolites. “Unknown” refers to metabolites with unknown biological origin

This study included 13 rabbits from a resilient and 13 rabbits from a non-resilient population, and for which 765 untargeted metabolites were identified from their guts. The Bayesian statistical analysis identified 66 metabolites with relevant differences (> 0.67 SD units) in abundance between the two populations (see Additional file 2: Table S1). The PLS-DA model for these 66 metabolites showed a prediction performance of 71 and 90% for the non-resilient and resilient animals, respectively. The most representative pathways were the long-chain fatty acylcarnitines (LCFA) metabolism, histidine metabolism, endocannabinoid metabolites, glycine, serine, and threonine metabolism, and tryptophan metabolism (Fig. 1a) and (see Additional file 2: Table S1). Of these 66 relevant metabolites, 29% were associated with a co-metabolism because they can be produced by both the host and the microbiota, 12% were associated with the microbiota (de novo synthesis), 27% were associated with other sources (24% food related and 3% drug related), and 32% could not be traced back to their origin (Fig. 1b).

**Fig. 2:**
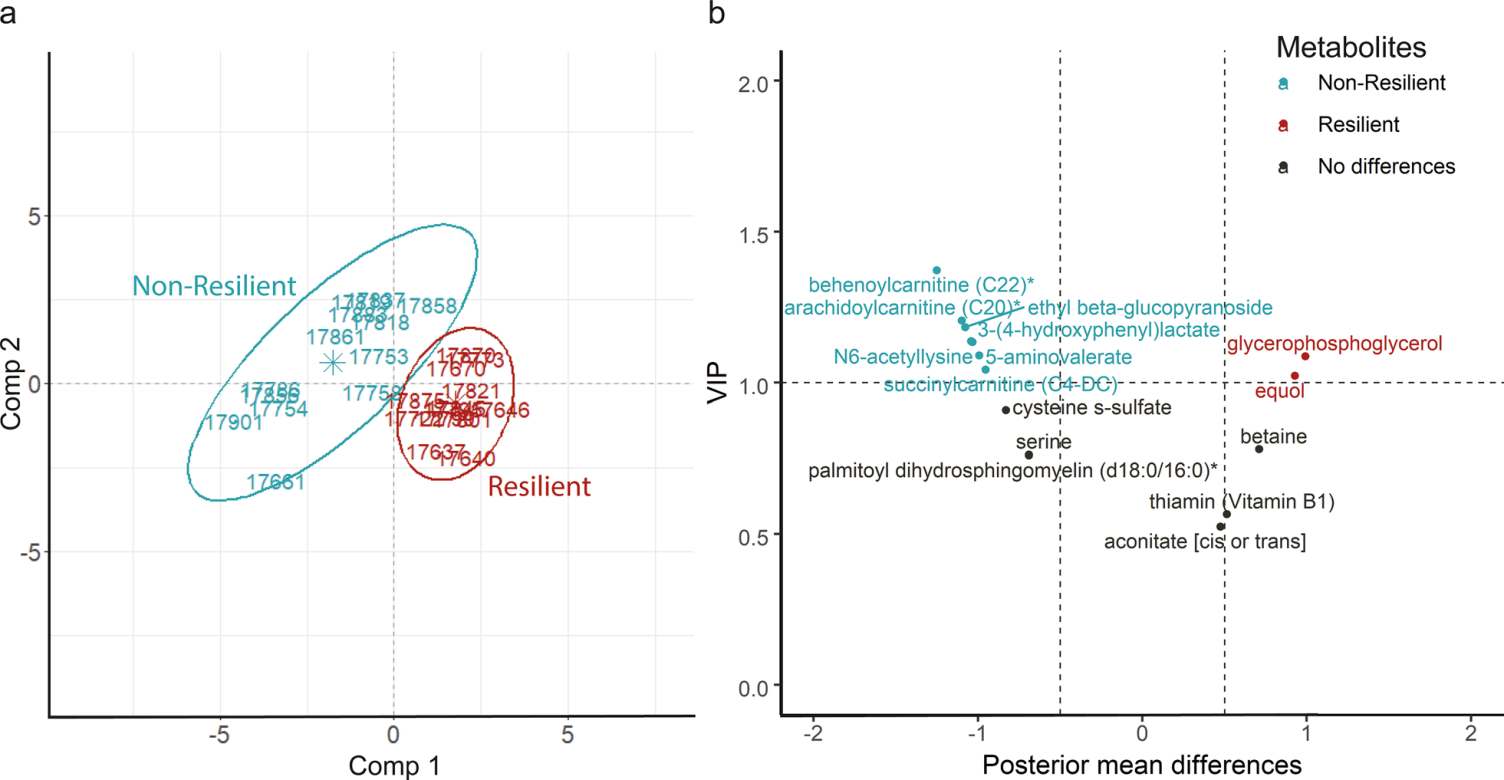
Final PLS-DA model. **a** Representation of the first (Comp 1) and second (Comp 2) components of the PLS-DA used to discriminate rabbits from the resilient (red) and non-resilient (blue) populations. **b** Representation of posterior mean differences in units of standard deviation (SD) and variable importance on prediction (VIP) of metabolites included in the final PLS-DA model. Blue dots are relevant metabolites identified as more abundant in the non-resilient population. Red dots are the relevant metabolites identified with greater abundance in the resilient population. Black dots are metabolites included in the final PLS-DA model but that did not exceed both VIP > 1 and a posterior mean of the differences > 0.5 SD

We also performed an optimized PLS-DA to identify the most relevant metabolites, i.e. those that reached the highest prediction performance, and found 15 metabolites with a prediction performance of 99.2 and 90.4% for, respectively, animals from the resilient and the non-resilient populations (Fig. 2a), i.e. behenoylcarnitine (C22), arachidoylcarnitine, ethyl beta-glucopyranoside, 3-(4-hydroxyphenyl)lactate, 5-aminovalerate, glycerophosphoglycerol, succinylcarnitine, equol, cysteine s-sulfate, betaine, serine, palmitoyl dihydrosphingomyelin, thiamine, and aconitate. These 15 metabolites are proposed as potential resilience biomarkers due to the low error achieved by the model to predict the divergent population that they belonged to. Thirteen of these 15 metabolites matched with those that were identified in the Bayesian analysis as differing in abundance between the rabbits from the divergent populations (see Additional file 2: Table S1). The two non-overlapping metabolites, aconitate and thiamine, showed the lowest contribution to the optimized PLS-DA model (Fig. 2b) and a difference in abundance of 0.5 units of SD. Of the 13 most reliable metabolites, five appeared to be derived from the diet (behenoylcarnitine, arachidoylcarnitine, succinylcarnitine, betaine, and palmitoyl dihihydrosphingomyelin), three from the microbiota (3-(4-hydroxyphenyl)lactate, 5-aminovalerate, and equol), and two from the co-metabolism between the host and the microbiota (N6-acetyllysine, and serine). For the remaining three metabolites, ethyl beta-glucopyranoside, glycerophosphoglycerol, and cysteine s-sulfate, no origin was determined.

## Discussion

The study of the gut metabolome can help unravel its effects on key traits in livestock. In this study, we found differences in the metabolite profile (see Additional file 2: Table S1) between rabbits from divergent populations for V_E_ of LS with differences in resilience potential [12]. The Bayesian analysis identified 66 metabolites with differences in abundance between rabbits from the divergent populations and good PLS-DA prediction performance to classify population origin. However, 13 of these 66 metabolites achieved the highest prediction performance to classify the resilient from the non-resilient animals. Hence, these metabolites were proposed as potential biomarkers for resilience. Our study also showed that 27 of the 66 metabolites and five of the 13 candidate resilience biomarkers (see Additional file 2: Table S1) originated from the microbiota (Fig. 1b). These results suggest that the microbiome composition differs between the two rabbit populations, in agreement with a previous metagenomic study for genes and taxa using the same populations [18]. In addition, we found that 16 of the 66 metabolites and (5 of the 13 candidate resilience biomarkers (see Additional file 2: Table S1) showed a biological origin related to the diet (Fig. 1b). These results suggest that the rabbits from the resilient and non-resilient populations differ in their feeding behaviour and/or the use of dietary compounds, either because of the host itself or their microbiota.

Relevant functions were identified for four of the five resilience biomarkers that were related to microbiota-derived metabolites (equol, 3-(4-hydroxyphenyl)lactate, 5-aminovalerate, N6-acetyl lysine, and serine). Equol (0.93 SD unit difference between the two populations), which derives from the daidzein metabolism, can develop neuroprotective effects [31] and trigger an immune response since it enhances macrophages and protects from oxidative stress [32]. As the rabbits were sacrificed after their first parity, the high levels of equol in the resilient animals may have helped to reduce the inflammatory response triggered by farrowing, which is a highly stressful event for the dam. Farrowing may also have increased the levels of the 3-(4-hydroxyphenyl)lactate biomarker (− 1.04 SD units difference) in the rabbits from the non-resilient population, which is a metabolite that derives from the degradation of tyrosine and has been associated with non-alcoholic hepatic liver diseases [33]. This metabolite could be involved in a gut-liver crosstalk based on differences found in the plasma levels of cholesterol and triglycerides between the animals of these two populations (after a challenge) [12, 14]. The 5-aminovalerate and N6-acetyl lysine metabolites are products of the degradation of lysine (KEGG ID: C00431); 5-aminovalerate in the presence of betaine, which is another resilience biomarker identified in our study (Fig. 2b) acts as a methyl substrate donor to form 5-aminovaleric acid betaine [34]. 5-aminovaleric acid betaine may not be identified correctly, thus its role in health and disease is still unclear (see Haikonen et al. [34] for more information). We did not find any evidence for the implication of N6-acetyl lysine in pathways related to animal resilience. However, catabolism of the identified resilience biomarker serine (-0.69 unit of SD) was suggested to be related to adaptation of pathogens during the inflammation process [35]. To support the relevance of this pathway, glycine, an interconverted molecule to serine (KEGG ID: C00037), was identified with a difference of − 0.68 SD unit between the two populations. Although it is not known how the serine levels act in the non-resilient population, it would be relevant to study its implication in animal resilience given its role in modulating bacterial pathogenesis.

We also identified other metabolites derived from the aromatic amino acids (AAA) metabolism (such as the abovementioned 3-(4-hydroxyphenyl) lactate) (Table 1). This is consistent with the differences in AAA biosynthesis genes (chorismite mutase and lyase) found in a previous metagenomic study using the same rabbit populations [18]. AAA metabolisms can control health and disease [36], by acting directly on the gut or on distal organs (i.e. liver, kidney or brain) [37], as well as modulate inflammatory bowel [37–39] and liver diseases such as hepatic inflammation and steatosis [37, 40]. Our results showed high levels of kynurenine and anthranilate (Table 1) in the rabbits from the non-resilient population, which showed higher levels of CRP (an inflammation biomarker) [12]. High levels of these two metabolites were also found in individuals that were under stress with inflammation [41]. As the animals were sacrificed after their first parity, the higher levels of kynurenine and anthranilate in the animals from the non-resilient population could pinpoint higher susceptibility to stress and inflammation in this population. Unexpectedly, the level of indole was found to be lower in the resilient rabbits (Table 1). This metabolite has protective functions in the gut by maintaining the intestinal barrier integrity and immune homeostasis, thus limiting dysbiosis during an inflammation response [42, 43]. An in-depth study would be needed to understand the role of the metabolites derived from the tryptophan metabolism on animal resilience.

**Table 1:** Metabolites from the aromatic amino acids (AAA) metabolism that had relevant differences between the non-resilient and resilient populations

Pathway	Metabolite	µ_H-L_	P_0_	HPD95
Tyrosine metabolism	3-(4-hydroxyphenyl)lactate	− 1.04	99.67	[− 1.55, − 0.22]
Phenylalanine metabolism	*N*-acetylphenylalanine	− 0.82	98.17	[− 1.57, − 0.04]
	Phenyllactate	0.71	96.38	[− 0.04, 1.52]
Tryptophan metabolism	Kynurenine	− 0.79	97.80	[− 1.55, − 0.22]
	Anthranilate	− 0.75	97.27	[− 1.51, 0.04]
	Oxindolylalanine	− 0.74	96.91	[− 1.51, 0.03]
	Indole	− 0.69	95.77	[− 1.49, 0.10]

µ_H-L_: posterior mean of the differences between the non-resilient and resilient populations

P_0_: probability of the difference being greater (if the difference is positive) or less (if negative) than 0

HPD95: high posterior density interval of 95%

The metabolites behenoylcarnitine, arachidoylcarnitine, steroylcarnitine, palmitoylcarnitine, and formiminoglutamate, were also highlighted. Behenoylcarnitine and arachidoylcarnitine were identified as potential resilience biomarkers by PLS-DA, while the other three only showed relevant differences in their abundance between the divergent populations (see Additional file 2: Table S1). The first four metabolites are long-chain fatty acylcarnitines (LCFA), which are biomarkers of gut dysbiosis [44] and it has been shown that high levels of LCFA in the gut are a biomarker for inflammatory bowel diseases due to mitochondrial dysfunction in the colonocytes [45]. Correct integrity and functionality of the intestinal epithelial barrier and colonocytes are essential to gut immunity homeostasis and pathogenesis [46–48]. These results suggest that differences in the assimilation of long-chain fatty acids by the gut for energy purposes could influence gut integrity and immunity. The fifth metabolite, formiminoglutamate, belongs to the histidine catabolism to l-glutamate pathway. Lower abundance of this metabolite was found in the resilient animals (see Additional file 2: Table S1), which is in line with a previous metagenomic study that reported higher levels of glutamate formiminotransferase in animals from the resilient population [18]. Our findings suggest that there are differences in l-glutamate metabolism between the two rabbit populations. Glutamate is an important neurotransmitter that can act in the gut, spinal cord, and brain, participates in the gut-brain axis, and influences inflammatory response [49].

For the potential resilience biomarker metabolites palmitoyl dihydrosphingomyelin, ethyl beta glucopyranoside, glycerophosphoglycerol, and cysteine-s-sulfate (Fig. 2b), no hypotheses about biological mechanisms affecting animal resilience can be made because their effects on the host are still unclear. However, we suggest that these metabolites are important for predicting and classifying the rabbits into the two rabbit divergent populations.

## Conclusions

This is the first study to identify gut metabolites that could act as potential biomarkers for resilience. Our results agree with the differences in resilience potential of these two rabbit populations, which were generated by divergent selection for V_E_ of LS. These differences could be due to the levels of acylcarnitines and of metabolites derived from amino acid metabolism, such as aromatic amino acids (tryptophan, phenylalanine and tyrosine), glycine, serine, and glutamate metabolism. Moreover, relevant metabolites, such as 3-(4-hydroxyphenyl)lactate could be involved in host-gut microbiota crosstalk. Selection for environmental variance has modified the gut metabolome, which could thus be another contributor to the differences in resilience between the rabbits from these divergent populations. However, further studies are needed to properly determine the origin and mode of action of each metabolite to unravel their causal role in health and disease, as well as in host-gut microbiota crosstalk.

## Supplementary Information

**Additional file 1.** Full pipeline with all the metabolomic analyses.

**Additional file 2: Table S1.** Results of the Bayesian statistical analysis. The file includes (from left to right) the metabolite ID, posterior mean of the differences among the resilient and non-resilient rabbit populations (meanDiff), the probability of the difference being higher (if the difference is positive) or lower (if negative) than 0 (P0), the highest posterior interval density of 95% (HPD95), the chemical name of the metabolites and the general and specific pathways in which they are involved, the posterior error probability (PEP), the cumulative PEP, the metabolites identified by the Bayesian analysis (Bayes), the metabolites identified by the PLS-DA (PLS), and the biological origin determined for each metabolite.

## Data Availability

Data are available upon request to the corresponding author.
