# Supplementary material for: Effect of environmental variance-based resilience selection on the gut metabolome of rabbits
Source: Genet Sel Evol. 2023 Mar 9;55:15. doi: 10.1186/s12711-023-00791-5 (PMC9996918; doi:10.1186/s12711-023-00791-5)
Supplement: Supplementary file 1 — Additional file 1. Full pipeline with all the metabolomic analyses. [file 12711_2023_791_MOESM1_ESM.html]

Metabonomic Analysis


# Metabonomic Analysis

#### Cristina Casto-Rebollo

#### 2022-05-04

- 1 Filtering variables with a high number of zeros
- 2 Removing outliers animals
- 3 Selection reference variable
- 4 Procrustes analysis
- 5 Additive log-ratio
- 6 Partial Least Square-Discriminant Analysis (PLS-DA)
  - 6.1 Quality of the model
    - 6.1.1 Confusion matrix
    - 6.1.2 Permutation matrix
- 7 Bayesian statistics Analysis
  - 7.1 Running the model
  - 7.2 Analyzing the differences among populations
  - 7.3 Volcano plot

```
# Library
library(pacman)
pacman::p_load(readxl,data.table,easyCODA,compositions,mixOmics,factoextra,dplyr,missForest,caTools,gtools,tidyverse,brms,ggridges,bayesplot,tidybayes,ggmcmc,bayestestR,ggrepel)

# Datasets

AUC<-data.frame(read_xlsx("UHMD-01-20VW DATA TABLE.XLSX",sheet = "Peak Area Data"))
ID<-read_xlsx("UHMD-01-20VW DATA TABLE.XLSX",sheet = "Sample Meta Data")
ANN<-read_xlsx("UHMD-01-20VW DATA TABLE.XLSX",sheet = "Chemical Annotation")

row.names(AUC)<-ID$SUBJECT_OR_ANIMAL_ID[match(AUC$PARENT_SAMPLE_NAME,ID$PARENT_SAMPLE_NAME)]
AUC<-AUC[,-1]
```

# 1 Filtering variables with a high number of zeros

```
# Population assignment

line<-ID$GROUP_NAME[match(rownames(AUC),ID$SUBJECT_OR_ANIMAL_ID)]

# Remove all variables with a percentage of zeros higher than 20% within-population

n.l <- round(length(which(line=="Low line"))*0.2)
n.h <- round(length(which(line=="High line"))*0.2)

zero.col<-data.frame(table(which(is.na(AUC),arr.ind = T)[,2]))
zero.col<-as.numeric(as.character(zero.col$Var1[zero.col$Freq>(n.l+n.h)]))

index.zero<-NULL;n<-0
for(i in zero.col){
  nh<-length(which(is.na(AUC[line=="High line",i])))
  nl<-length(which(is.na(AUC[line=="Low line",i])))
  
  if(nh/nl>0.5 & nh/nl<2){
    n<-n+1
    index.zero[n]<-i
  }
}

indx.l <- which(is.na(AUC[line=="Low line",]),arr.ind = T)
indx.h <- which(is.na(AUC[line=="High line",]),arr.ind = T)
zeros.l <- data.frame(table(indx.l[,2]))
zeros.h <- data.frame(table(indx.h[,2]))

indx.l <- as.numeric(as.character(zeros.l$Var1[which(zeros.l$Freq>=n.l)]))
indx.h <- as.numeric(as.character(zeros.h$Var1[which(zeros.h$Freq>=n.h)]))

col <- unique(c(indx.h[indx.h%in%indx.l],index.zero))

length(col)
```

```
## [1] 111
```

```
AUC.zero <- AUC[,-col]
```

# 2 Removing outliers animals

```
##Remove all zeros in the dataset
zero.col<-data.frame(table(which(is.na(AUC),arr.ind = T)[,2]))
zero.col<-as.numeric(as.character(zero.col$Var1[zero.col$Freq>0]))
AUC.noZero<-AUC[,-zero.col]

## Principal Component Analysis

pca<-prcomp(AUC.noZero,scale=T)
fviz_pca_ind(pca,geom=c("point","text"),col.ind=line,axes=c(1,2),addEllipses = TRUE,ellipse.level = 0.95,pointsize=1)
```

```
indx<-grep("17763|17808",rownames(AUC.noZero))
AUC.noZero<-AUC.noZero[-indx,]

##Repetition PCA

pca<-prcomp(AUC.noZero,scale=T)
fviz_pca_ind(pca,geom=c("point","text"),col.ind=line[-indx],axes=c(1,2),addEllipses = TRUE,ellipse.level = 0.95,pointsize=1)
```

# Zero imputation

Imputation using the half of minimum value of intensity peak detected per each metabolite.

```
##Imputation with minimum intensity peak
AUC.zero<-AUC.zero[-indx,]
line<-line[-indx]
line<-gsub(" line","",line)


zero.col<-data.frame(table(which(is.na(AUC.zero),arr.ind = T)[,2]))
zero.col<-as.numeric(as.character(zero.col$Var1[zero.col$Freq>0]))

AUC.imp<-AUC.zero
for(i in zero.col){
  AUC.imp[is.na(AUC.imp[,i]),i]<-min(AUC.imp[,i],na.rm = T)/2
}
```

# 3 Selection reference variable

```
n <- dim(AUC.imp)[2]
cv <- data.frame(Variable=numeric(n),CV=numeric(n))
cv$Variable <- colnames(AUC.imp)
for (i in 1:n) {
  
  cv$CV[i] <- sd(log(AUC.imp[,i])) / mean(log(AUC.imp[,i]))
  cv$Total[i] <- sum(AUC.imp[,i])
  cv$mean[i] <- mean(AUC.imp[,i])
  }

cv <- cv[order(cv$CV,decreasing=F),]
cv$Variable[1]; cv$CV[1]; cv$mean[1]
```

```
## [1] "X825"
```

```
## [1] 0.01141287
```

```
## [1] 16940322
```

```
ref <- cv$Variable[1]

indx.ref <- grep(ref,names(AUC.imp))
```

# 4 Procrustes analysis

Procrustes analysis was performed to test if the selected reference variable allowed how close they come to the exact geometry. This means that the variables maintain their relationship despite the transformation.

Procrustes analysis is based on three simple operations:

- Centering
- Scaling
- Rotation

```
LRA <- LRA(AUC.imp,weight = F)$rowpcoord

ALR <- ALR(AUC.imp,denom = indx.ref)$LR
ALR.PCA <- PCA(ALR,weight=F)$rowpcoord

print(paste("Procruste correlation:",round(protest(LRA,ALR.PCA[,1:ncol(LRA)], permutations=0)$t0,2)))
```

```
## [1] "Procruste correlation: 0.99"
```

# 5 Additive log-ratio

Calculation of the additive log-ratio of each variable and centering and scaling them.

```
AUC.ALR <- log(AUC.imp/AUC.imp[,indx.ref])
AUC.ALR <- AUC.imp[,-indx.ref]

AUC.center <- AUC.ALR
for (i in 1:ncol(AUC.ALR)) {
  
  AUC.center[,i] <- AUC.ALR[,i] - mean(AUC.ALR[,i])
  AUC.center[,i] <- AUC.center[,i] / sd(AUC.center[,i])

}
```

# 6 Partial Least Square-Discriminant Analysis (PLS-DA)

The PLS-DA tries to extract the latent structures (patterns) that allow explaining a dependent variable (Y; response). With this analysis, we can reduce the dimension of the data and collect the information throughout the maximization of the covariance between the X and Y. In this case, X is the ALR matrix with all KEGG and Y is a vector with the Line/Population of each sample.

```
#Computing the PLS-DA model with 10 components
plsda <- plsda(AUC.center,line,ncomp=10,scale=F)

#4-fold cross-validation to compute the balance error rate (BER)

set.seed(30)
perf.pls <- perf(plsda, validation = "Mfold",criterion="all",folds = 4,
                 progressBar = F,nrepeat = 100,)

#Optimal number of components

comp <- perf.pls[["choice.ncomp"]][6]

#BER for the number of components selected

err.n <- perf.pls[["error.rate"]]$BER[20+comp]
sd.n <- perf.pls[["error.rate.sd"]]$BER[20+comp]
err.total <- err.n + sd.n

#Variable important prediction (VIP). Contribution of each variable in the classification among lines/populations
vip <- data.frame(vip(plsda),stringsAsFactors = FALSE)

#A VIP higher than 1 was used as a threshold for selecting the variables with the highest contribution in the model

p <- 1
v.select <- vip[vip[,comp]>=p,]
v.ID <- row.names(v.select)

#Iterative process to obtain the best model with the lower BER

err <- 1
comp.cte <- 10

while (err.total<err) {
  
  err <- err.total
  sd.f <- sd.n
  comp.f <- comp
  
  vf<-v.ID
  vip <- data.frame(vip(plsda),stringsAsFactors = FALSE)
  v.select <- vip[vip[,comp] >= p,]
  
  v.ID <- row.names(v.select)
  
  if (length(v.ID) == 1) {
      break
  
    } 
  
  filter <- AUC.center[,names(AUC.center) %in% v.ID]
  
   if (length(v.ID)<comp.cte) {
    
    comp.cte <- length(v.ID)
  
  }
  
  plsda <- plsda(filter,line,ncomp=comp.cte,scale=F)
  set.seed(30)
  perf.pls <- perf(plsda, validation = "Mfold",criterion="all",folds = 4,
                   progressBar = F,nrepeat = 100,)

  
  comp <- perf.pls[["choice.ncomp"]][6]
  err.n <- perf.pls[["error.rate"]]$BER[(comp.cte*2)+comp]
  sd.n <- perf.pls[["error.rate.sd"]]$BER[(comp.cte*2)+comp]
  err.total <- err.n + sd.n
}
```

In summary, the model with the minimum BER (Balance error rate) for the classification will be the following specifications:

```
#Optimal number of components in the model

comp.f
```

```
## [1] 2
```

```
#Balanced error rate of the model

err - sd.f
```

```
## [1] 0.04846154
```

```
#Standard deviation of the BER

sd.f
```

```
## [1] 0.03122238
```

```
#Number of variables included in the model

length(vf)
```

```
## [1] 15
```

```
#Relevant variables for the classification

write.table(names(AUC[grep(paste(vf,collapse = "|"),names(AUC))]),"C:/Users/3ccas/OneDrive - UPV/2020_2021/Metabolomica/Result_final/relevant_metabolites.txt",row.names = T,col.names = T,quote = F,sep="\t")

#Final model

filter <- AUC.center[,names(AUC.center) %in% vf]
line2<-line
line2[line2=="Low"]<-"Resilient";line2[line2=="High"]<-"Non-Resilient"

#PLS-Plot of the final model
color <- c("#31A2AC","#AF1C1C","#2F2F28","#F0EFF0")

if (comp.f > 1) {
  
  plsda <- plsda(filter,line2,ncomp=comp.f,scale=F)

  plotIndiv(plsda,ind.names = TRUE, legend=TRUE,style = "ggplot2",rep.space = "X-variate",
             ellipse = TRUE, centroid=TRUE,title = 'Final PLS-DA model',
             X.label = 'Comp 1', Y.label = 'Comp 2',col = color[1:2],abline=TRUE,xlim = c(-9,9),cex = c(5,5),point.lwd = 1,
             ylim=c(-9,9),
             size.title = rel(2), size.subtitle = rel(2), size.xlabel = rel(2),
             size.ylabel = rel(2), size.axis = rel(1.5), size.legend = rel(2),
             size.legend.title = rel(2.2),
             legend.title = "Population",alpha=1)+ theme_classic()
}
```

```
## NULL
```

```
#PCA-Plot of final model
pca <- prcomp(filter,scale=F)
fviz_pca_ind(pca,axes=c(1,2),geom = c("point","text"),col.ind=line2,addEllipses = T,palette=color,ellipse.level = 0.95,pointsize = 1)
```

```
p<-fviz_pca_ind(pca,axes=c(1,2),geom = c("point","text"),col.ind=line2,addEllipses = T,palette=color,ellipse.level = 0.95,pointsize = 1)+
  geom_point(aes(fill=line2,color=line2,shape=line2))+
  scale_shape_manual(values=c(16,17))+
  scale_color_manual(values =color)+
  scale_fill_manual(values =color) +
  xlim(-9, 9) + ylim (-9, 9)+
  geom_hline(yintercept = 0, colour="#2F2F28", linetype="dashed") + 
  geom_vline(xintercept = 0, colour="#2F2F28", linetype="dashed") + 
  labs(x ="PC1 (38.7%)", y = "PC2 (13.7%)",title = NULL,face="bold",fill="Population",shape="Population",color="Population") + 
  theme_minimal() +
  theme_classic()+
  theme( 
    legend.position="bottom",
    panel.border = element_blank(),
    panel.grid.major.x = element_blank(),
    panel.grid.minor.x = element_blank(),
    axis.text.x = element_text( size = 9, vjust = 1.5),
    axis.text.y = element_text( size = 9, vjust = 0.7))


ggsave("Result_final/pca_relevant.tiff",p, width = 100, height = 80, units = "mm",dpi = 600)

plsda <- plsda(filter,line,ncomp=comp.f,scale=F)
vip <- data.frame(vip(plsda),stringsAsFactors = FALSE)

write.table(vip[,1:comp.f],"C:/Users/3ccas/OneDrive - UPV/2020_2021/Metabolomica/Result_final/VIP.txt",sep = "\t",quote=F)
write.table(filter,"C:/Users/3ccas/OneDrive - UPV/2020_2021/Metabolomica/Result_final/Fullrel_metabolites.txt",row.names = T,col.names = T,quote = F,sep="\t")
```

## 6.1 Quality of the model

To check the quality of the model, two tests were performed using a 4-fold Cross-validation 10,000 times specifying the Mahalanobis distance

### 6.1.1 Confusion matrix

Allow knowing the success rate for the prediction of each rabbit line/population. Percentage of false positive and false negative.

```
data.RF <- cbind(filter,line)
confusion.total <- matrix(ncol=2,nrow=2,0)
x.total <- NULL
for (i in 1:10000) {
  
  sample = sample.split(data.RF$line, SplitRatio = .70)
  train = subset(data.RF, sample == TRUE)
  test  = subset(data.RF, sample == FALSE)
  dim(train)
  dim(test)
  
  x <- data.frame(table(test$line))
  x.total <- rbind(x,x.total)
  plsda.train <- plsda(train[,-ncol(train)],train$line,ncomp=comp.f,scale=F)
  test.predict <- predict(plsda.train,test[,-ncol(test)],dist = "mahalanobis.dist")
  prediction <- test.predict$class$mahalanobis.dist[,comp.f]
  
  confusion.mat <- get.confusion_matrix(truth = test$line,predicted =prediction )
  confusion.total <- confusion.total + confusion.mat

  }

High <- sum(x.total$Freq[x.total$Var1 == "High"])
Low <- sum(x.total$Freq[x.total$Var1 == "Low"])

confusion.total[1,] <- 100 * confusion.total[1,] / High
confusion.total[2,] <- 100 * confusion.total[2,] / Low

confusion.total
```

```
##      predicted.as.High predicted.as.Low
## High           90.4200           9.5800
## Low             0.8225          99.1775
```

### 6.1.2 Permutation matrix

Allow computing the spurious rate for the random prediction of each rabbit line/population.

```
cross.val <- data.RF
permutation.total <- matrix(ncol=2,nrow=2,0)
x.total <- NULL
for (i in 1:10000) {
  
  data.RF$line <- permute(cross.val$line)
  sample = sample.split(data.RF$line, SplitRatio = .70)
  train = subset(data.RF, sample == TRUE)
  test  = subset(data.RF, sample == FALSE)
  dim(train)
  dim(test)
  
  x <- data.frame(table(test$line))
  x.total <- rbind(x,x.total)
  plsda.train <- plsda(train[,-ncol(train)],train$line,ncomp=comp.f,scale=F)
  test.predict <- predict(plsda.train,test[,-ncol(test)],dist = "mahalanobis.dist")
  prediction <- test.predict$class$mahalanobis.dist[,comp.f]
  
  confusion.mat <- get.confusion_matrix(truth = test$line,predicted =prediction )
  permutation.total <- permutation.total + confusion.mat

  }

High <- sum(x.total$Freq[x.total$Var1=="High"])
Low <- sum(x.total$Freq[x.total$Var1=="Low"])

permutation.total[1,] <- 100 * permutation.total[1,] / High
permutation.total[2,] <- 100 * permutation.total[2,] / Low

permutation.total
```

```
##      predicted.as.High predicted.as.Low
## High            49.970           50.030
## Low             50.225           49.775
```

# 7 Bayesian statistics Analysis

Bayesian statistics were used to compute the relevance/importance of each variable for the differentiation among the rabbit populations. A good introduction to the Bayesian data analysis can find in Blasco, A. Bayesian Data Analysis for Animal Scientists: The Basics. 2017. Springer, Cham.

DOI: https://doi.org/10.1007/978-3-319-54274-4

```
line<-as.factor(line)
data.RF <- cbind(filter,line)
#Number and position of treatment variables

treat<-1
pT<-ncol(data.RF)

#Number and position of noising variables

noise <- 0
pNoise <- NA

#Number of response variables

nResp <- ncol(data.RF) - noise - treat
```

## 7.1 Running the model

```
prior <- set_prior("normal(0,1)",class="b")
samples <- NULL

set <- ceiling(nResp / 100)

group <- round(nResp / set)  
init = -group+2
end = 0
for (n.set in 1:set) {
    
  init = init+group
  end = end + group
    
  if (n.set == set) {

    end <- nResp
  
    }
    
  brm.equation <- list()
  for (var in init:end) {
  
     brm.equation <- append(brm.equation,(formula(paste(names(data.RF[var]),"~",names(data.RF)[pT],collapse = ""))))

     }

  eq.multiple <- mvbf(bf(formula(paste(names(data.RF[init-1]),"~",names(data.RF)[pT],collapse = ""))),flist = brm.equation,rescor = F)

  model <- brm(eq.multiple,
           data = data.RF, 
           family = gaussian(), iter = 50000, chains = 4, warmup = 1000,thin=10 ,control = list(adapt_delta = 0.99),
           silent=2,refresh=0,backend = "cmdstanr",
           threads = threading(7))
  
  conver<-data.frame(summary(model)$fixed)
  conver$convergence<-0
  conver$convergence<-ifelse(conver$Rhat<1.05 &conver$Rhat>0.95,"OK","FAIL")
  
  modelfit= as.data.frame(fitted(model,
                               newdata = expand.grid(line= levels(data.RF[,pT])), re_formula = NA, summary = FALSE))

  if (n.set == 1) {
    conver.tmp<-conver
    model.fit<-modelfit
    
    } else {
      
    conver.tmp<-rbind(conver.tmp,conver)
    model.fit<-cbind(model.fit,modelfit)
  
    }
}
```

```
## Running MCMC with 4 sequential chains, with 7 thread(s) per chain...
## 
## Chain 1 finished in 247.5 seconds.
## Chain 2 finished in 212.6 seconds.
## Chain 3 finished in 227.2 seconds.
## Chain 4 finished in 219.1 seconds.
## 
## All 4 chains finished successfully.
## Mean chain execution time: 226.6 seconds.
## Total execution time: 908.4 seconds.
```

## 7.2 Analyzing the differences among populations

We computed the differences among the average of the mcmc chains of the variables for each population. The difference was calculated substrating the values of the chains from the high population to the low population (Low - High). The result showed the mean difference among the populations as differences in standard deviation. Moreover, we calculated the probability of the difference to be higher or lower than zero (P0). The highest posterior density interval of 95% was also computed. The most relevant variables were the variables with a P0 higher than 0.9 (90%) and a mean difference higher than 0.5 of SD.

```
print(paste("Convergence not reached:",which(conver.tmp$convergence=="FAIL"),sep=" "))
```

```
## [1] "Convergence not reached: "
```

```
colnames(model.fit) <- gsub("1\\.","High.",names(model.fit))
colnames(model.fit) <- gsub("2\\.","Low.",names(model.fit))

Low <- names(model.fit)[grep("Low",names(model.fit))]
High<-names(model.fit)[grep("High",names(model.fit))]

m<-length(Low)
result<-data.frame(Metabolite=character(m),meanDiff=numeric(m),P0=numeric(m),HPD95=numeric(m))

for (i in 1:m) {
  
  result$Metabolite[i] <- gsub("X","",names(data.RF)[i])
  
  sample.diff <- model.fit[,names(model.fit) == Low[i]] - model.fit[,names(model.fit) == High[i]]
  
  hdi <- hdi(sample.diff,ci=0.95)
  
  result$HPD95[i] <- paste("[",round(hdi$CI_low,2),",",round(hdi$CI_high,2),"]",sep = "")
  
  if (mean(sample.diff) > 0) {
    
    result$P0[i] <- 100 * (1 - ecdf(sample.diff)(0))
  
    } else {
    
      result$P0[i] <- 100 * ecdf(sample.diff)(0)
  
      }
  
  result$meanDiff[i] <- mean(sample.diff)

}

result$CHEMICAL_NAME<-ANN$CHEMICAL_NAME[match(result$Metabolite,ANN$CHEM_ID)]
result$SUPER_PATH<-ANN$SUPER_PATHWAY[match(result$Metabolite,ANN$CHEM_ID)]
result$SUB_PATH<-ANN$SUB_PATHWAY[match(result$Metabolite,ANN$CHEM_ID)]

knitr::kable(result, format="markdown")
```

| Metabolite | meanDiff | P0 | HPD95 | CHEMICAL\_NAME | SUPER\_PATH | SUB\_PATH |
| --- | --- | --- | --- | --- | --- | --- |
| 240 | -1.0319548 | 99.72449 | [-1.73,-0.33] | 3-(4-hydroxyphenyl)lactate | Amino Acid | Tyrosine Metabolism |
| 503 | -0.6895949 | 95.87755 | [-1.46,0.11] | serine | Amino Acid | Glycine, Serine and Threonine Metabolism |
| 799 | 0.7116321 | 96.53061 | [-0.07,1.48] | betaine | Amino Acid | Glycine, Serine and Threonine Metabolism |
| 873 | 0.5143965 | 89.93367 | [-0.29,1.35] | thiamin (Vitamin B1) | Cofactors and Vitamins | Thiamine Metabolism |
| 100000454 | -0.9921895 | 99.55612 | [-1.7,-0.26] | 5-aminovalerate | Amino Acid | Lysine Metabolism |
| 100000808 | -0.8287358 | 98.22449 | [-1.57,-0.05] | cysteine s-sulfate | Amino Acid | Methionine, Cysteine, SAM and Taurine Metabolism |
| 100001359 | 0.4758732 | 87.60204 | [-0.33,1.31] | aconitate [cis or trans] | Energy | TCA Cycle |
| 100001619 | 0.9919303 | 99.56633 | [0.26,1.7] | glycerophosphoglycerol | Lipid | Glycerolipid Metabolism |
| 100001725 | 0.9294990 | 99.19898 | [0.18,1.66] | equol | Xenobiotics | Food Component/Plant |
| 100001734 | -1.0413611 | 99.69388 | [-1.73,-0.32] | N6-acetyllysine | Amino Acid | Lysine Metabolism |
| 100001948 | -0.9531145 | 99.35714 | [-1.69,-0.21] | succinylcarnitine (C4-DC) | Energy | TCA Cycle |
| 100008954 | -0.6904967 | 96.00510 | [-1.47,0.08] | palmitoyl dihydrosphingomyelin (d18:0/16:0)\* | Lipid | Dihydrosphingomyelins |
| 100015832 | -1.2512270 | 99.95408 | [-1.9,-0.6] | behenoylcarnitine (C22)\* | Lipid | Fatty Acid Metabolism (Acyl Carnitine, Long Chain Saturated) |
| 100015833 | -1.0979451 | 99.79592 | [-1.82,-0.41] | arachidoylcarnitine (C20)\* | Lipid | Fatty Acid Metabolism (Acyl Carnitine, Long Chain Saturated) |
| 100020377 | -1.0773958 | 99.79592 | [-1.78,-0.37] | ethyl beta-glucopyranoside | Xenobiotics | Food Component/Plant |

```
write.table(result,"C:/Users/3ccas/OneDrive - UPV/2020_2021/Metabolomica/Result_final/result_bayes_relevant.txt",row.names = F,quote=F, sep="\t")
```

## 7.3 Volcano plot

Volcano plot was made using the posterior mean of the differences among the marginal distribution of the rabbit populations.

```
#VIP of each variable
result$VIP<-vip$comp1[match(paste0("X",result$Metabolite,sep=""),rownames(vip))]


mycolors <- c("#31A2AC","#AF1C1C","#2F2F28")
names(mycolors) <- c("Non-Resilient", "Resilient", "No differences")

result$diffexpressed <- "No differences"

# if meanDiff > 0.5 and pvalue < 0.05, set as "Resilient" 
result$diffexpressed[result$meanDiff >= 0.5 & result$VIP >= 1] <- "Resilient"
# if meanDiff < -0.5 and pvalue < 0.05, set as "Non-Resilient"
result$diffexpressed[result$meanDiff <= -0.5 & result$VIP >= 1] <- "Non-Resilient"


result$delabel<-result$CHEMICAL_NAME
result$delabel[!paste0("X",result$Metabolite,sep="")%in%vf]<-NA

volcano<-ggplot(data=result, aes(x=meanDiff, y=VIP,col=diffexpressed,label=delabel)) + geom_point(size=1)+
  theme_classic()+
  scale_colour_manual(values = mycolors)+
  geom_vline(xintercept=c(-0.5,0.5), col="#2F2F28",linetype="dashed",size=0.3) +
  geom_hline(yintercept=1, col="#2F2F28",linetype="dashed",size=0.3)+
  labs(x ="Posterior mean differences", y = "VIP")+
  xlim(-2,2)+
  ylim(0,2)+
  theme(legend.title = element_blank(),legend.position = c(0.80,0.9),
        axis.text.x = element_text( size = 9, vjust = 1.5),
        axis.text.y = element_text( size = 9, vjust = 0.7))+
  geom_text_repel(size=3,max.overlaps = 10,    box.padding = unit(0.1, "lines"),
    point.padding = unit(0, "lines"))


volcano
```

```
ggsave("Result_final/volcano_relevant.tiff",volcano, width = 120, height = 110, units = "mm",dpi = 600)
```
